# Supplementary figures and images for: Preservation of developmental spontaneous activity enables early auditory system maturation in deaf mice
Source: PLoS Biol. 2023 Jun 27;21(6):e3002160. doi: 10.1371/journal.pbio.3002160 (PMC10298803; doi:10.1371/journal.pbio.3002160)

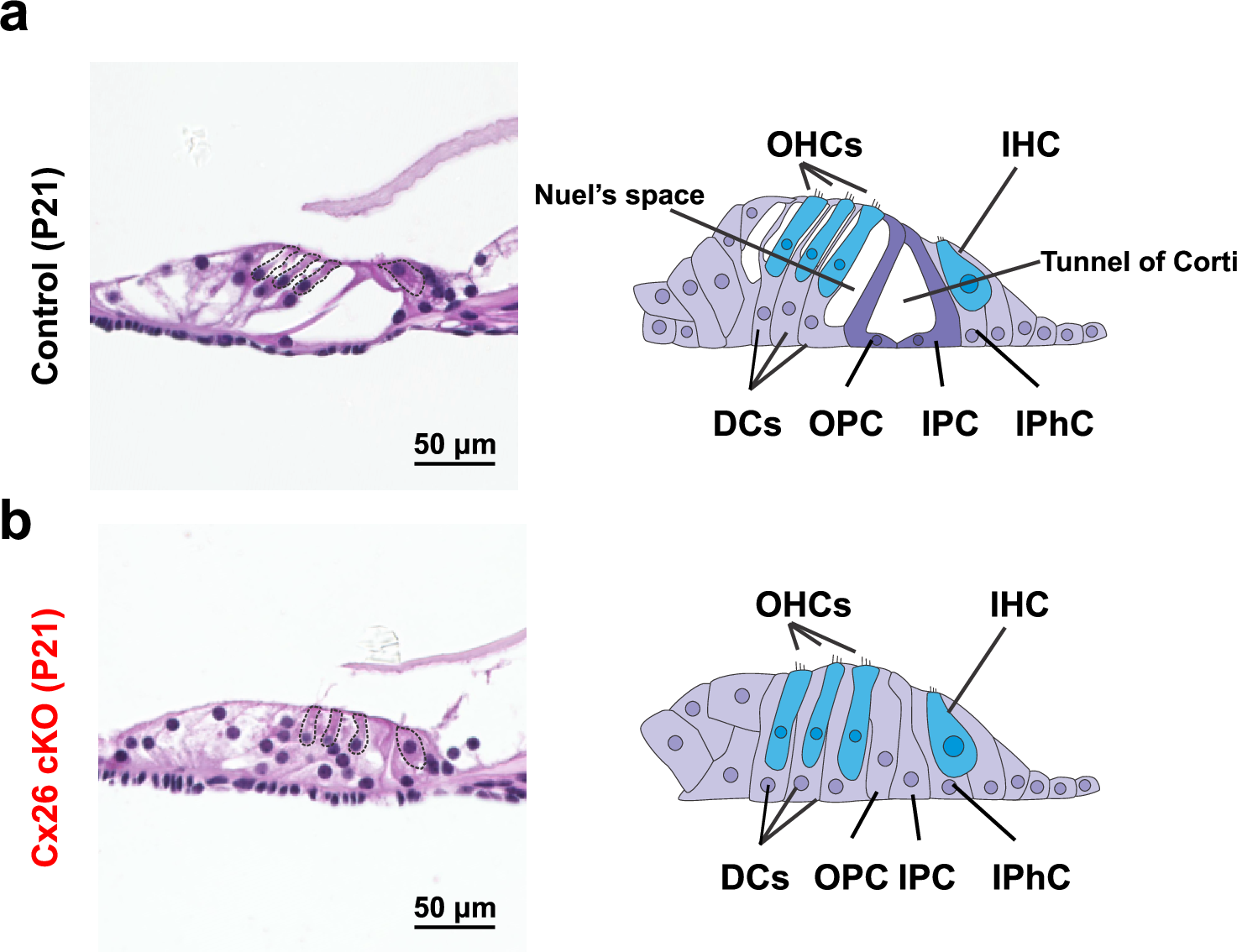

Supplement: S1 Fig — (a) (Left) Hematoxylin and eosin stain of a P21 control (Gjb2fl/fl) organ of Corti at high magnification. Hair cell soma are outlined by dashed black lines. (Right) Schematic depicting morphology of hair cells and supporting cells in the control organ of Corti. IPhC, inner phalangeal cell; IPC, inner pillar cell; OPC, outer pillar cell; DCs, Deiters’ cells; OHCs, outer hair cells; IHC, inner hair cell. (b) (Left) Hematoxylin and eosin stain of a P21 Cx26 cKO (Tecta-Cre;Gjb2fl/fl) organ of Corti at high magnification. Hair cell soma are outlined by dashed black lines. (Right) Schematic depicting morphology of hair cells and supporting cells in the Cx26 cKO organ of Corti. Analysis code, plotted figure panels, and statistical analysis can be found at: https://doi.org/10.5281/zenodo.7896212. (TIF) [file pbio.3002160.s001.tif]

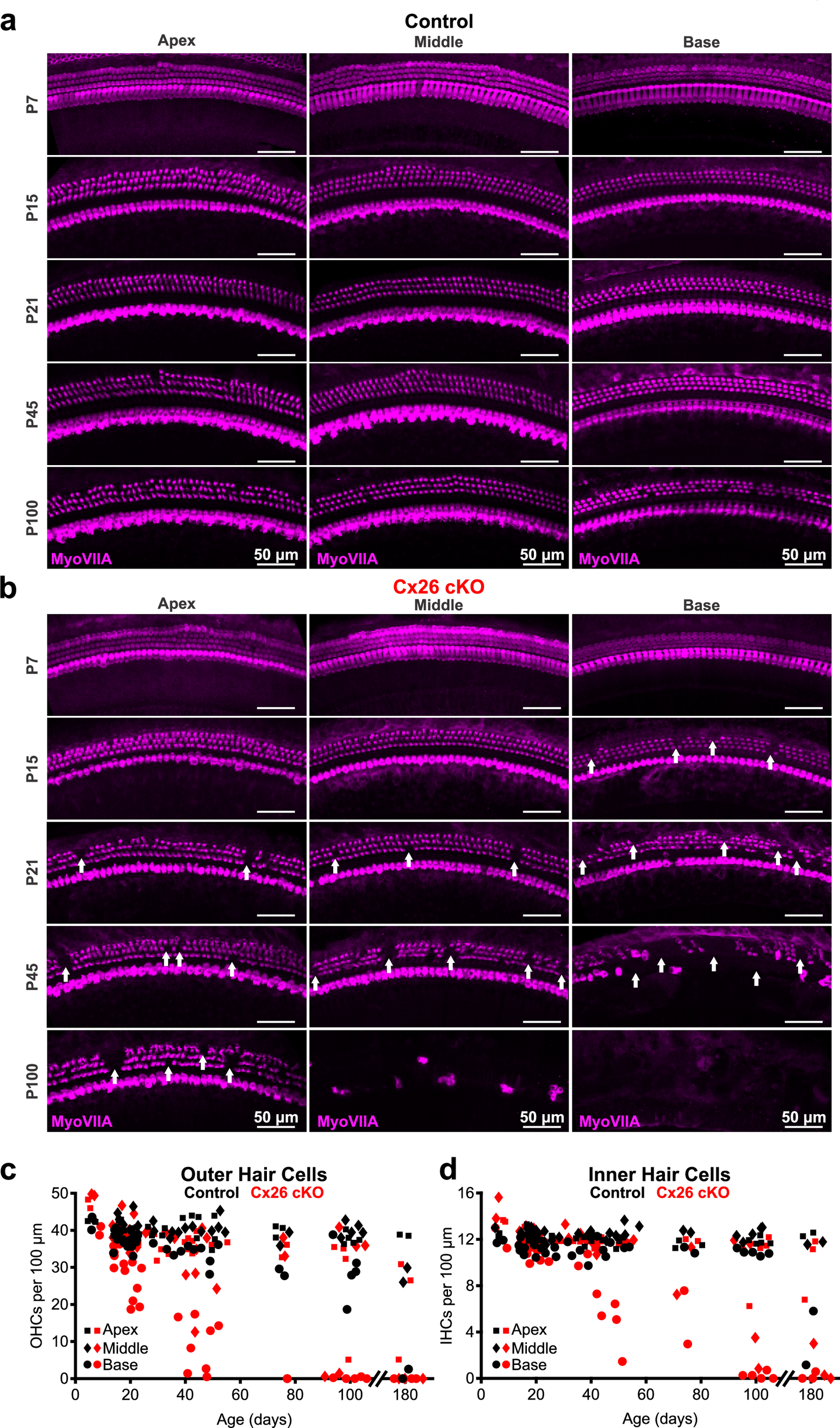

Supplement: S2 Fig — (a) Representative images of hair cells labeled by immunoreactivity to Myosin VIIa (magenta) in whole mounts from apex, middle, and basal regions in control (Gjb2fl/fl) cochleae at P7, P15, P21, P45, and P100. (b) Representative images of hair cells labeled by immunoreactivity to Myosin VIIa (magenta) in whole mounts from apex, middle, and basal cochlea regions in Cx26 cKO (Tecta-Cre;Gjb2fl/fl) cochleae at P7, P15, P21, P45, and P100. (c) Quantification of outer hair cells (OHCs, left) and inner hair cells (IHCs, right) with increasing age in apical (squares), middle (diamonds), and basal (circles) regions of control and Cx26 cKO cochlea. Analysis code, plotted figure panels, and statistical analysis can be found at: https://doi.org/10.5281/zenodo.7896212. (TIF) [file pbio.3002160.s002.tif]

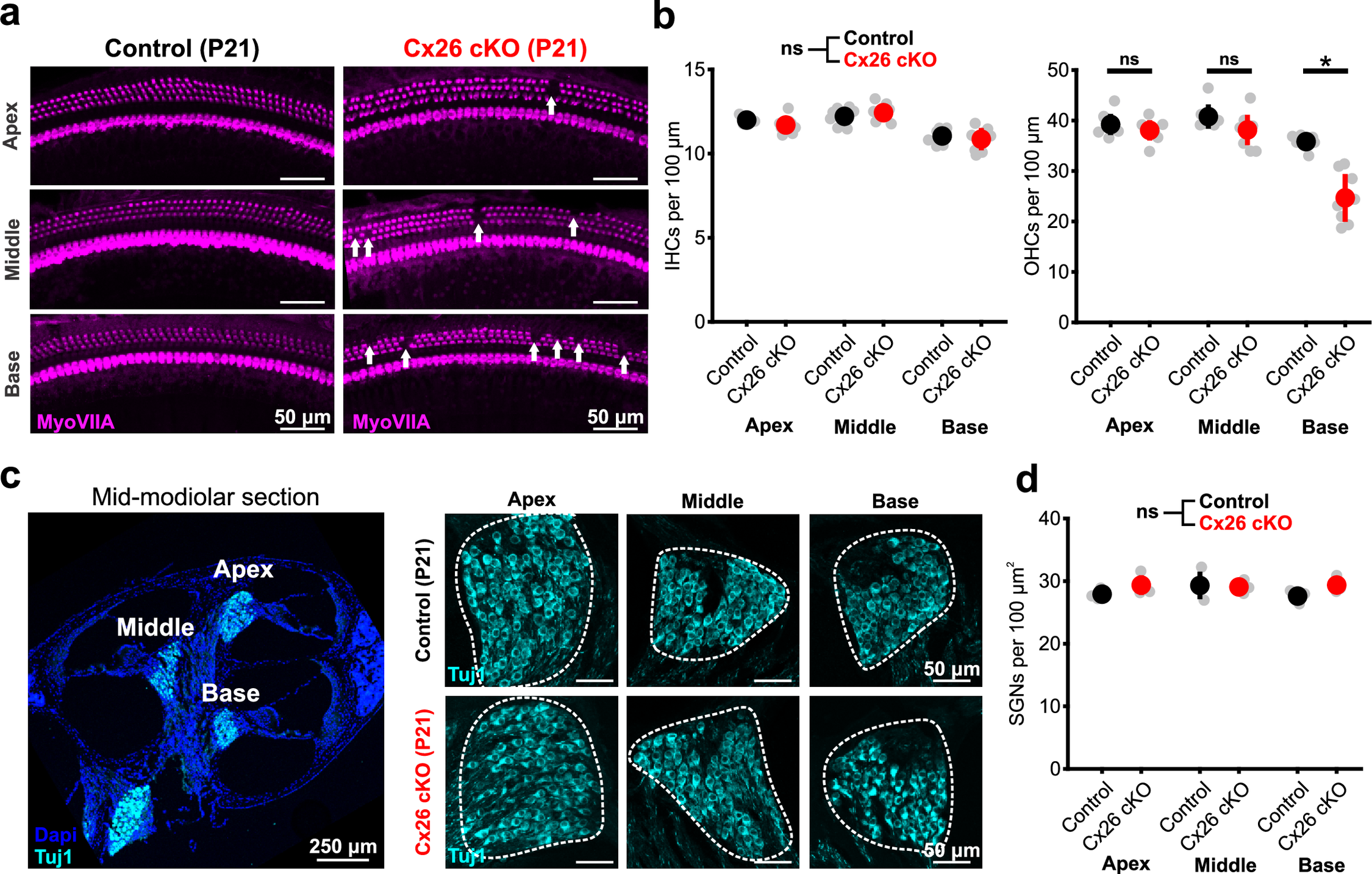

Supplement: S3 Fig — (a) Representative images of hair cells labeled by immunoreactivity to Myosin VIIa (magenta) in whole mounts from apex, middle, and basal cochlea in control (Gjb2fl/fl, left) and Cx26 cKO (Tecta-Cre;Gjb2fl/fl, right) mice at P21. White arrows indicate sites of outer hair cell loss. (b) Quantification of inner hair cells (IHCs, left) and outer hair cells (OHCs, right) at P21 in control and Cx26 cKO apical, middle, and basal cochlea segments. n = 8 control cochleae, 8 Cx26 cKO cochleae; p = 0.0974, 0.0021 (IHCs, OHCs), linear mixed model with Sidák post hoc test. (c) (Left) Low magnification image of SGNs labeled by immunoreactivity to Tuj1 (cyan) in mid-modiolar cross section of P21 cochlea. Labels indicate locations of apical, middle, and basal SGN counts. (Right) Representative high-magnification images of SGN soma labeled by immunoreactivity to Tuj1 in apical, middle, and basal cochlea from control and Cx26 cKO mice at P21. Dashed lines indicate SGN compartment used for area measurement. (d) Quantification of SGN density in apical, middle, and basal cochlea at P21. n = 3 control cochleae, 3 Cx26 cKO cochleae; p = 0.4385, repeated measures ANOVA with lower bound p value adjustment. Analysis code, plotted figure panels, and statistical analysis can be found at: https://doi.org/10.5281/zenodo.7896212. (TIF) [file pbio.3002160.s003.tif]

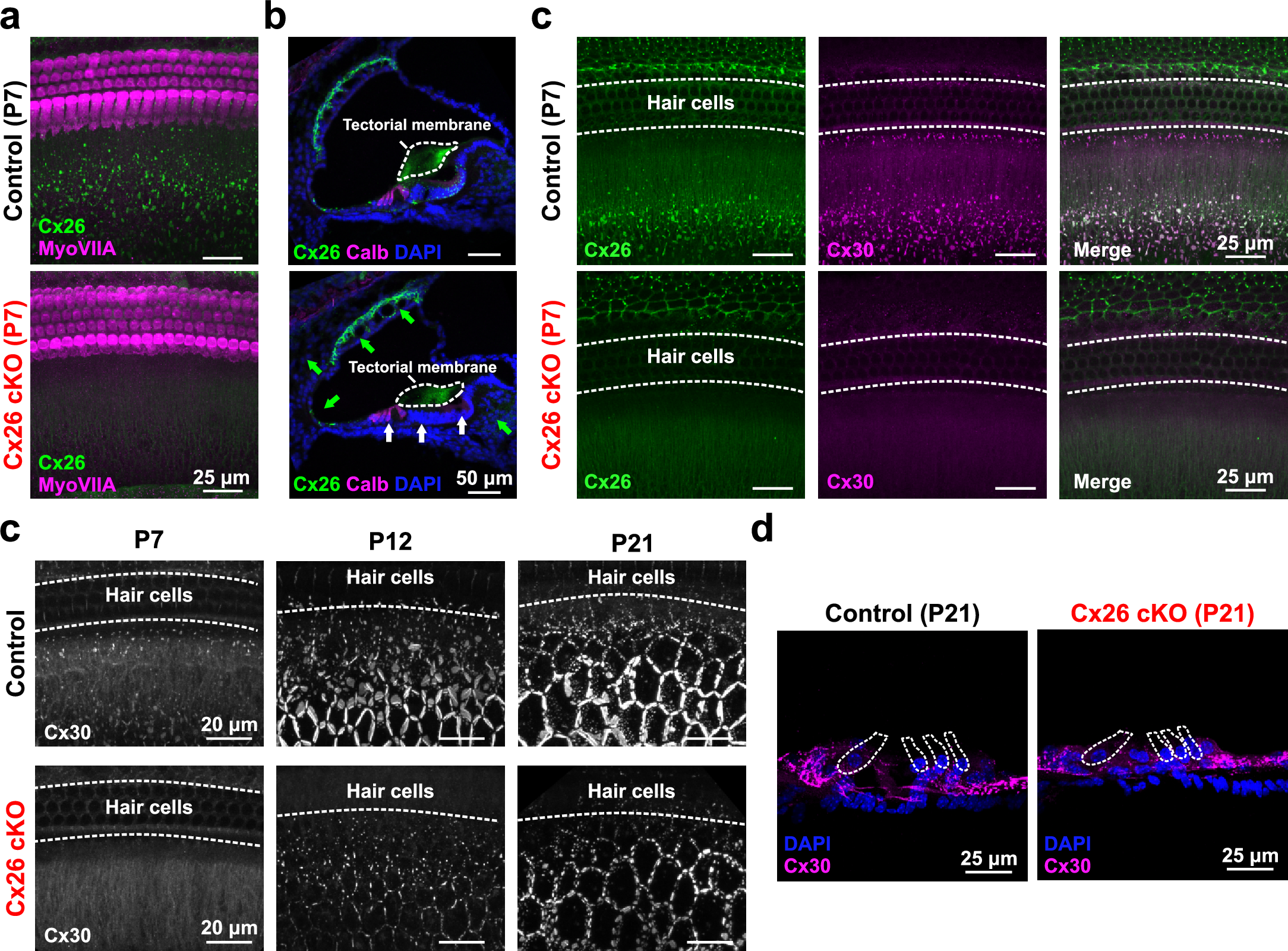

Supplement: S4 Fig — (a) Immunostaining for Connexin 26 (green) in whole mount middle P7 cochlea from control (Gjb2fl/fl) and Cx26 cKO (Tecta-Cre;Gjb2fl/fl) mice. Hair cells (magenta) are labeled by immunoreactivity to Myosin VIIA. (b) Immunostaining for Connexin 26 (green) in mid-turn cochlea cross section at P7. Loss of Cx26 immunostaining is observed in Kölliker’s organ and supporting cells of the organ of Corti (white arrows) but not within lateral wall or spiral limbus fibrocytes or the stria vascularis (green arrows). Hair cells are labeled by antibodies against Calbindin (magenta). (c) Immunostaining for Connexin 26 (green) and Connexin 30 (magenta) in whole mount P7 apical organ of Corti from control (top) and Cx26 cKO (bottom) mice. (d) Immunostaining for Connexin 30 (white) in inner supporting cells from whole mount cochlea across increasing developmental age (P7, P12, P21) in control (top) and Cx26 cKO (bottom) mice. (e) Immunostaining for Connexin 30 (magenta) in apical cross section of the organ of Corti at P21. White dashed lines indicate hair cell location. Analysis code, plotted figure panels, and statistical analysis can be found at: https://doi.org/10.5281/zenodo.7896212. (TIF) [file pbio.3002160.s004.tif]

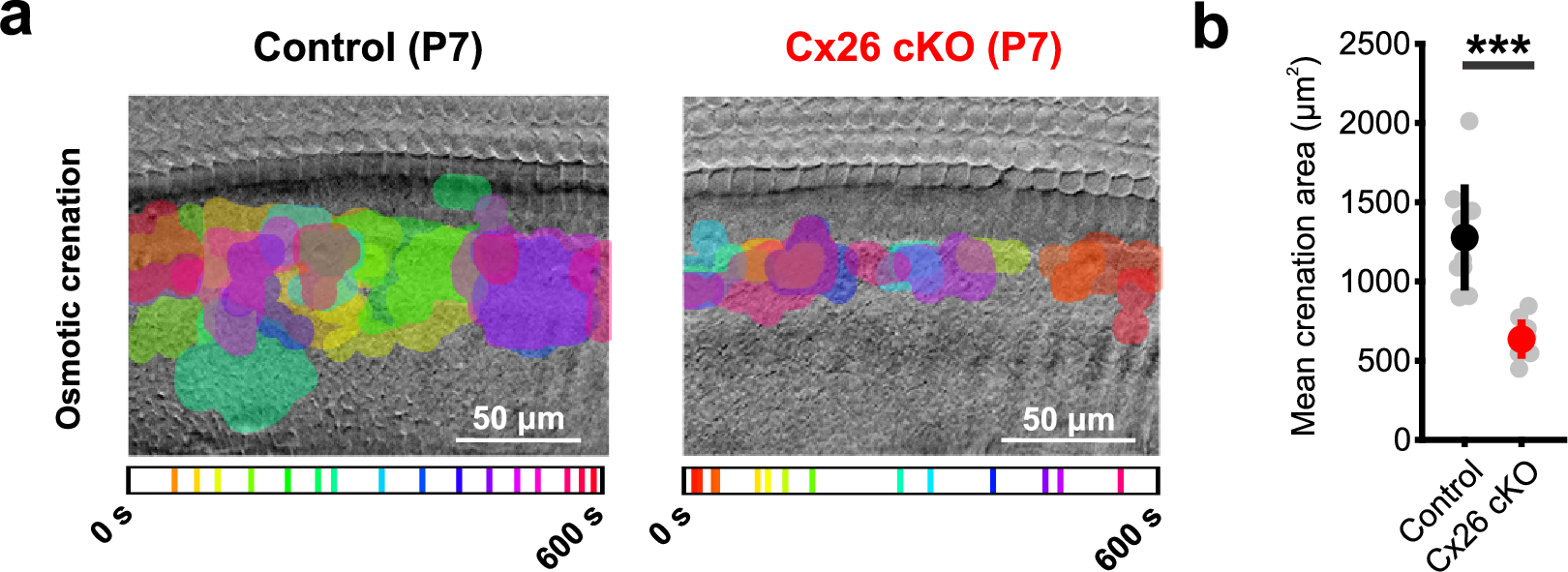

Supplement: S5 Fig — (a) Intrinsic optical imaging of osmotic crenations in control and Cx26 cKO cochleae. Detected crenations are indicated with transparent colored areas based on time of occurrence. (b) Quantification of spontaneous crenation area. n = 9 control, 9 Cx26 cKO cochleae; p = 4.4669e-4, two-sample t test with unequal variances. Analysis code, plotted figure panels, and statistical analysis can be found at: https://doi.org/10.5281/zenodo.7896212. (TIF) [file pbio.3002160.s005.tif]

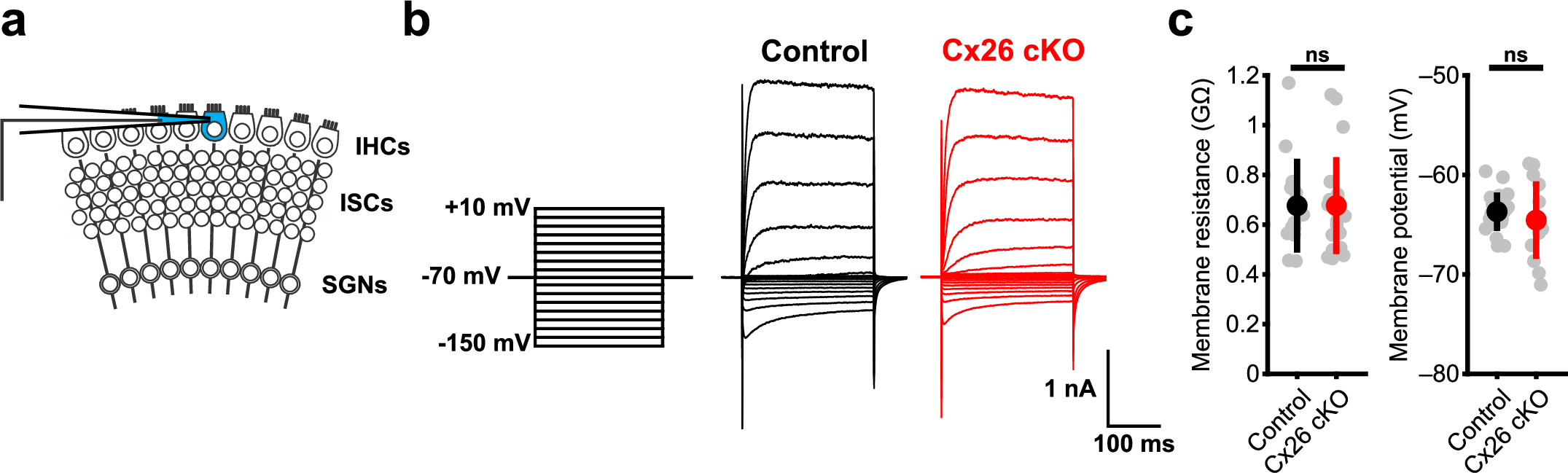

Supplement: S6 Fig — (a) Schematic of whole cell patch clamp recording from IHCs. (b) Voltage protocol (left) and representative current responses (right) from P7 control (Gjb2fl/fl) and Cx26 cKO (Tecta-Cre;Gjb2fl/fl) IHCs. (c) Quantification of membrane resistance and resting membrane potential in P7 control and Cx26 cKO IHCs. n = 13 control, 19 Cx26 cKO; p = 0.9838, 0.9847 (membrane potential, membrane resistance), two-sample t test (membrane potential) or Wilcoxon rank sum test (membrane resistance) with Benjamini–Hochberg correction. Analysis code, plotted figure panels, and statistical analysis can be found at: https://doi.org/10.5281/zenodo.7896212. (TIF) [file pbio.3002160.s006.tif]

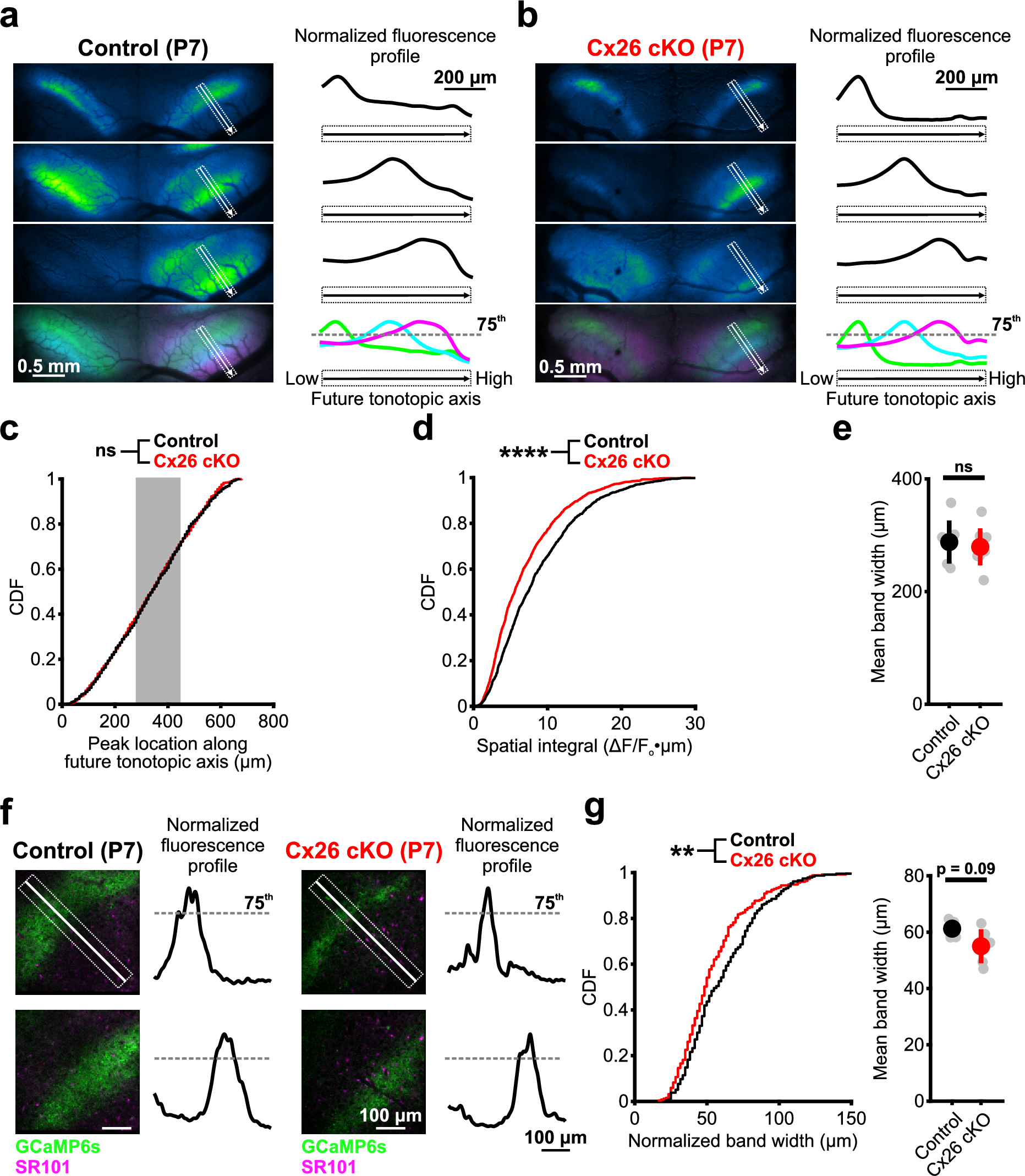

Supplement: S7 Fig — (a) Representative spontaneous neural calcium transients (left) and corresponding normalized spatial fluorescence profile along future tonotopic axis (right, indicated by white rectangle) in IC from P7 control (Gjb2fl/fl;Snap25-T2A-GCaMP6s) mouse. Merged pseudocolored image (bottom) highlights spatial segregation of discrete spontaneous calcium transients. Band width is calculated as the 75th percentile of the normalized spatial profile. (b) Same as (a), but from P7 Cx26 cKO (Tecta-Cre;Gjb2fl/fl;Snap25-T2A-GCaMP6s) mouse. (c) Cumulative distribution of spontaneous event peak location along tonotopic axis of the IC. Gray shading represents central tonotopic region (40th–60th percentile) used for calculation of band width in (e). n = 1,290 control events (7 mice), 1,385 Cx26 cKO events (8 mice); p = 0.4908, two-sample Kolmogorov–Smirnov test. (d) Quantification of spatial integral of all spontaneous events along tonotopic axis. n = 1,290 control events (7 mice), 1,385 Cx26 cKO events (8 mice); p = 1.0825e-9, two-sample Kolmogorov–Smirnov test. (e) (Left) Quantification of mean normalized band width (75th percentile) of spontaneous events along tonotopic axis within central IC. n = 7 control mice, 8 Cx26 cKO mice; p = 0.6620, Wilcoxon rank sum test. (f) Representative spontaneous calcium transients in neurons and neuropil within the inferior colliculus of P7 control (left) and P7 Cx26 cKO (right) mice using two-photon imaging. SR101 (magenta) labels astrocytes for image registration. Normalized fluorescence profile for each event was calculated. (g) (Left) Cumulative distribution of normalized band width of spontaneous calcium transients measured using two-photon imaging. n = 236 control events (5 colliculi, 3 mice), 253 Cx26 cKO events (5 colliculi, 3 mice); p = 0.0059, two-sample Kolmogorov–Smirnov test. (Right) Quantification of mean band width. n = 5 control colliculi (3 mice), 5 Cx26 cKO colliculi (3 mice); p = 0.0958, two-sample t test with unequal variances. Ana [file pbio.3002160.s007.tif]

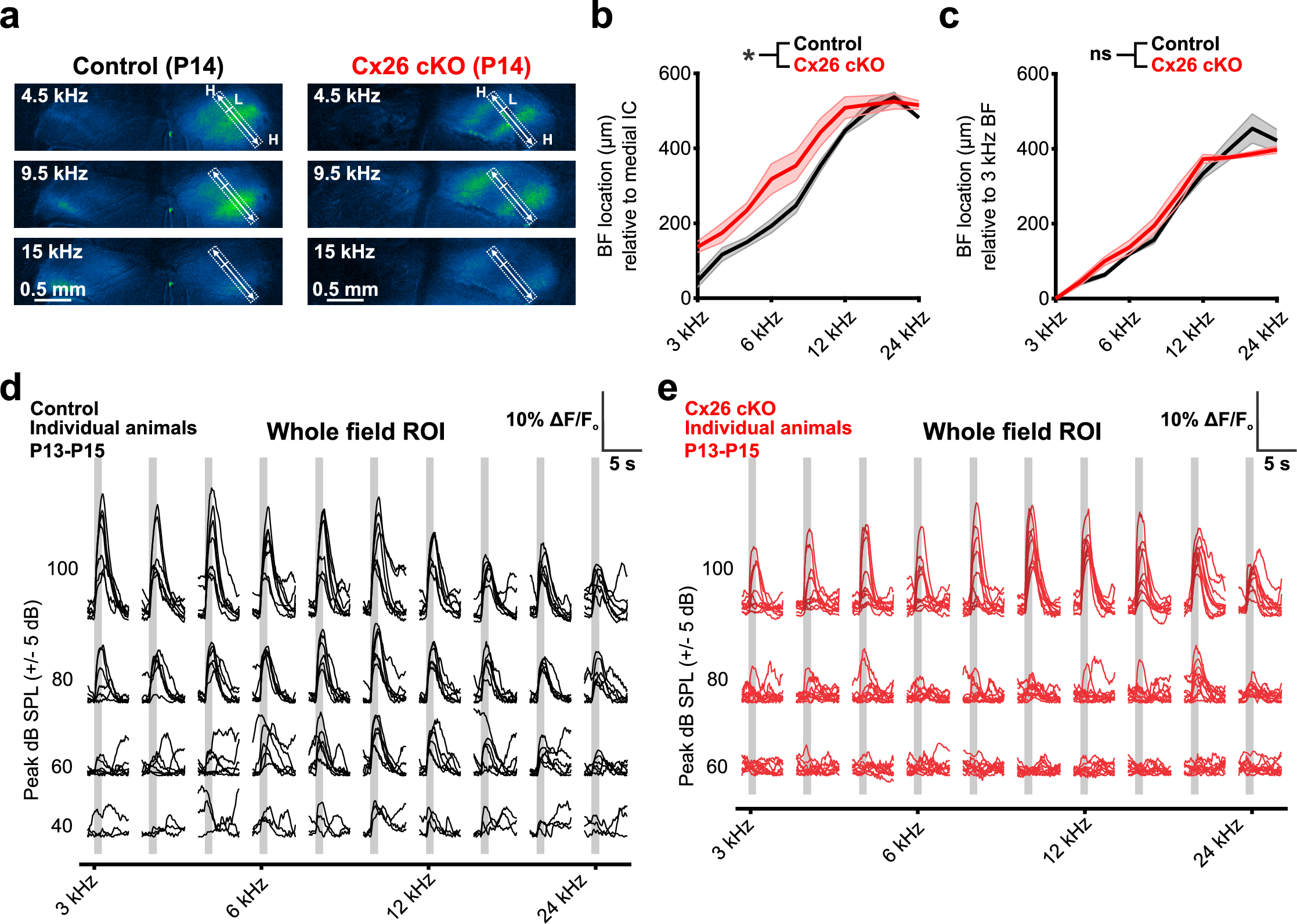

Supplement: S8 Fig — (a) Tone-evoked neural calcium transients in IC from P14 control (Gjb2fl/fl;Snap25-T2A-GCaMP6s, left) and P14 Cx26 cKO (Tecta-Cre;Gjb2fl/fl;Snap25-T2A-GCaMP6s, right) mice at 100 dB SPL. Rectangular ROIs were placed along the tonotopic axis of the contralateral IC (low (L) to high (H) frequency), perpendicular to pure tone evoked bands, to determine the peak response location for a pure tone along the tonotopic axis. (b) Quantification of peak response location of pure tones along the tonotopic axis relative to the medial IC. n = 4 control mice, 5 Cx26 cKO mice; mean ± SEM, p = 0.0205, linear mixed effects model. (c) Quantification of peak response location of pure tones along the tonotopic axis relative to 3 kHz (lowest frequency) peak response location. n = 4 control mice, 5 Cx26 cKO mice; mean ± SEM, p = 0.5654, linear mixed effects model. (d) Quantification of tone-evoked fluorescence changes in IC within whole IC ROIs across a range of frequency and sound level stimuli in individual P13-P15 control mice. Each black line indicates average responses from the IC contralateral to acoustic stimulation within an individual animal. Vertical gray bar indicates tone presentation. (e) Quantification of tone-evoked fluorescence in IC across a range of frequency and sound level stimuli in individual P13-P15 Cx26 cKO mice. Each red line indicates average responses from the IC contralateral to acoustic stimulation within an individual animal. Vertical gray bar indicates tone presentation. Analysis code, plotted figure panels, and statistical analysis can be found at: https://doi.org/10.5281/zenodo.7896212. (TIF) [file pbio.3002160.s008.tif]

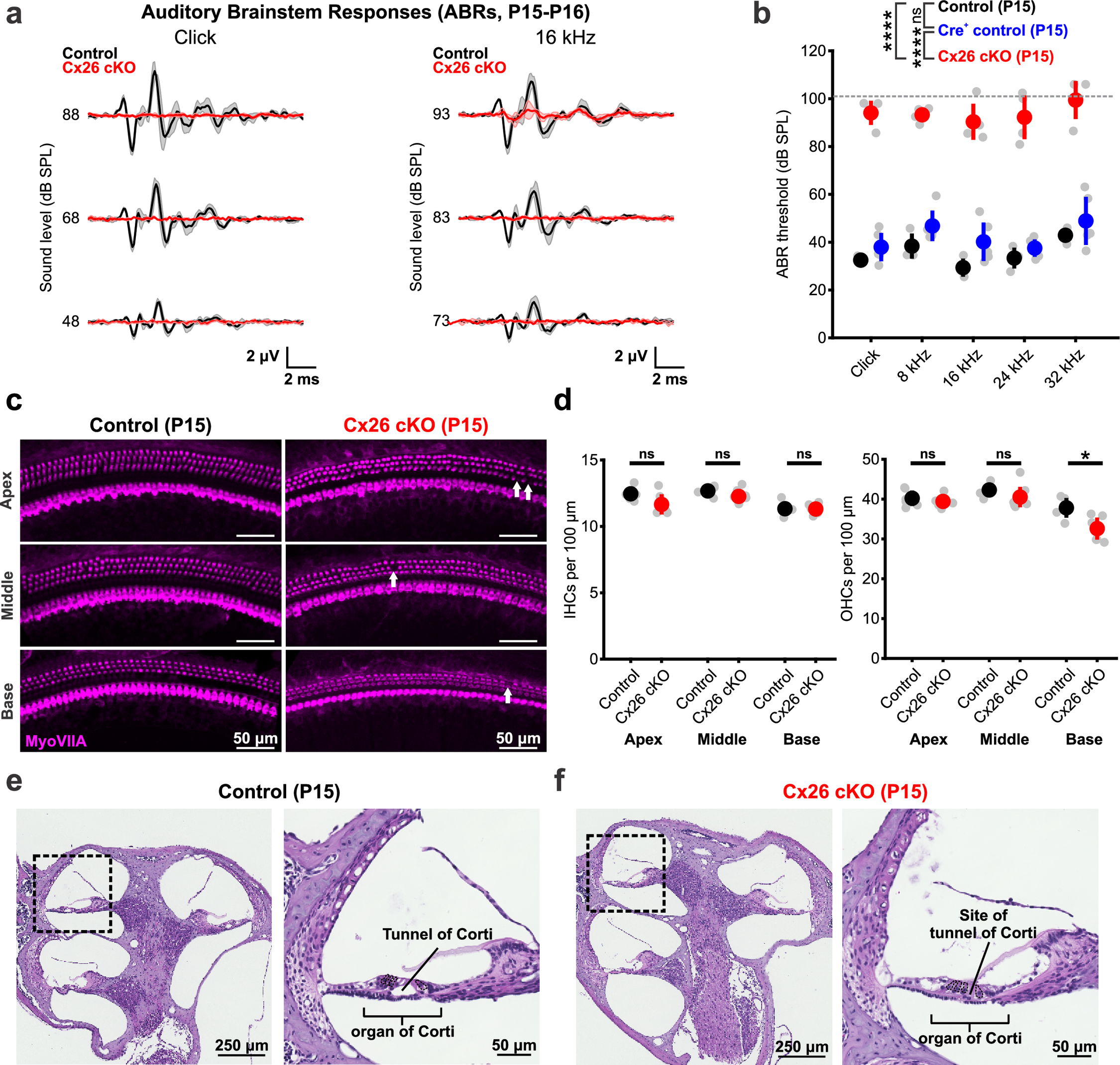

Supplement: S9 Fig — (a) Average ABR traces to broadband click (left) and 16 kHz tone pip stimuli at multiple sound pressure levels from control (Gjb2fl/fl, black, n = 3) and Cx26 cKO (Tecta-Cre;Gjb2fl/fl, red, n = 4) at P15-P16. Shaded region represents standard deviation of the average signals across animals. (b) Quantification of P15-P16 ABR thresholds to click and pure tone stimuli in controls (Gjb2fl/fl, black, n = 3 and Tecta-Cre;Gjb2fl/+, blue, n = 5) and Cx26 cKO (Tecta-Cre;Gjb2fl/fl, red, n = 4). Detection limit dashed line indicates maximum output from speaker. p = 4.4717e-9 (cKO vs. control), 8.6968e-6 (cKO vs. Cre+ control), 0.1454 (control vs. Cre+ control), linear mixed effects model. (c) Representative images of hair cells labeled by immunoreactivity to Myosin VIIa (magenta) in whole mounts from apex, middle, and basal cochlea in control (Gjb2fl/fl, left) and Cx26 cKO (Tecta-Cre;Gjb2fl/fl, right) at P15. White arrows indicate sites of outer hair cell loss. (d) Quantification of inner hair cells (IHCs, left) and outer hair cells (OHCs, right) at P15-P16 in control and Cx26 cKO apical, middle, and basal cochlea segments. n = 9 control, 9 Cx26 cKO cochleae; p = 0.0326, 0.0053 (IHCs, OHCs), linear mixed model with Sidák post hoc test. (e) (Left) Hematoxylin and eosin stain of a mid-modiolar section of P15 control cochlea. Black square indicates site of high magnification. (Right) Magnified image of the organ of Corti, with the tunnel of Corti indicated by black line. (f) (Left) Hematoxylin and eosin stain of a mid-modiolar section of P15 Cx26 cKO cochlea. Black square indicates site of high magnification. (Right) Magnified image of the organ of Corti, with site of the tunnel of Corti indicated by black line. Analysis code, plotted figure panels, and statistical analysis can be found at: https://doi.org/10.5281/zenodo.7896212. (TIF) [file pbio.3002160.s009.tif]
